# A Supplement to Self-Organization Theory of Dreaming

**DOI:** 10.3389/fpsyg.2016.00332

**Published:** 2016-03-08

**Authors:** Wei Zhang

**Affiliations:** School of psychology, Nanjing Normal UniversityNanjing, China

**Keywords:** self-organization, dreaming, memory consolidation, two-stage model, by-product

## Dreaming: A process of self-organization

Kahn and Hobson (1993) proposed that dreams are a product of self-organization of brain during sleep. As a complex system far from equilibrium state, the dreaming brain may form a new pattern by the interaction between components within this system. At REM sleep stage, signals from neuronal clusters self-organize and form image fragments, then the image fragments interact and produce images, and finally these materials are associated into a relatively continuous narrative (i.e., dreams).

This process above happens under a weak control of brain, and the conditions of this state mainly include: (a) the inhibition of external stimuli or feedback; (b) the changes of neuromodulatory systems (e.g., the governance of aminergic neurochemicals and the weakness of cholinergic neurochemicals); (c) the bombardment of PGO wave to the visual cortex; and (d) the changes of neural activity in brain (e.g., the activation of limbic system and the reduction of prefrontal regions). In this situation, the brain could focus on the internal world and integrate various psychological and physiological changes into dream content (Kahn et al., 2000, 2002).

However, self-organization mechanism could not determine which memories will be activated and incorporated into dream content (Kahn, 2013), although it provides an approach how an ordered structure, behavior or pattern spontaneously emerges from the interaction between components or subsystems without an external guidance (see Isaeva, 2012; Sasai, 2013). It merely combines materials which present during sleep and then makes up a “story.” Thus, the content of dreams is, to a large degree, determined by the active materials in sleep. In this article, we attempt to make a supplement to this theory on the basis of the two-stage model of memory consolidation.

## Dreaming and memory consolidation

Memory is a major element of dreams. The Continuity Hypothesis (CH) considers that the content of dreams is a reflection of waking life, and the former is also carried over into the latter (Domhoff, 1996). Thus, the episodic memories of real life will replay during sleep. This viewpoint has been supported by many investigations about dreams that contain waking experiences (e.g., Domhoff, 2001, 2003; Schredl and Piel, 2005; Pesant and Zadra, 2006).

However, there are several problems that the Continuity Hypothesis could not explain. Firstly, according to this theory, major daily activities, such as highly focused cognitive processes (e.g., writing, reading, and arithmetic), will present in dream content, but the consequence is not (e.g., Hartmann, 2000). Secondly, although experiences of waking life could be found in dreams, the incorporated components in dreams are usually fragments rather than a whole (Fosse et al., 2003). This means real life is not entirely reply in dreams (e.g., Schwartz, 2003; Hartmann, 2010). Hence, the Continuity Hypothesis seems not to be the ultimate answer.

An alternative explanation may be found in studies about the relationship between sleep and memory consolidation. According to the two-stage memory model, memory consolidation during sleep mainly contains a series of process as follows (McClelland et al., 1995; Nielsen and Stenstrom, 2005; Diekelmann and Born, 2010; Lewis and Durrant, 2011; Born and Wilhelm, 2012). (a) Newly acquired memories will be reactivated in NREM sleep, especially in SWS (Born and Wilhelm, 2012; Wamsley, 2014). In this period, there will be a mechanism that selects and determines which memories should be strengthened or weakened. If these memories are useful for the individual, they will be enhanced. If not (i.e., these memories are non-adaptive), they will be eliminated or fade away (Stickgold and Walker, 2013). Thus, this stage which mainly occurs in hippocampus will refer to a procedure of abstraction and extraction, and it leads to fragments of memory. (b) The temporary stored memories in hippocampus will be transferred into long-term memory which stores in neocortex about 6–7 days later. This stage needs to integrate these temporary memories into existing schema. Thus, it is associated with a course of redistributing memories and usually results in new connections (Hartmann, 2010).

During these processes, the role of NREM sleep and that of REM sleep are not the same; they seem to be complementary (Diekelmann and Born, 2010; Rasch and Born, 2013). Furthermore, these two stages of sleep are involved with different types of memory. For instance, NREM sleep is more closely related to declarative memory, while REM sleep is mostly correlated with emotional memory and memory that participates in implicit learning (Rauchs et al., 2005; Smith, 2010). This could be a reason why dream content in NREM sleep is different from that in REM sleep.

Therefore, memories will be fragmentized in order to extract important information (e.g., newly encoded and emotional memories) during sleep, and these important information are preferentially activated and incorporated into dream content. They mainly contain: (a) new learning experiences (Born and Wilhelm, 2012; Wamsley, 2014) which need to be consolidated and will be involved with a process of reactivation. Evidences could be found in many studies about dream reports (e.g., Stickgold et al., 2000; Wamsley et al., 2010a,b) and preferential reactivation of corresponding brain regions (e.g., Peigneux et al., 2004; Rasch et al., 2007; Rudoy et al., 2009; O'Neill et al., 2010; Bendor and Wilson, 2012) after learning tasks; (b) memories that will be integrated into long-term memory. This procedure is mainly reflected in dream-lag effect in REM sleep (Nielsen et al., 2004; Nielsen and Stenstrom, 2005; Blagrove et al., 2011; van Rijn et al., 2015); (c) long-term memories that are compatible with newly stored memories. As the former has to make connections with the latter in order to form a new cognitive schema (Lewis and Durrant, 2011), this process will be involved with remote memories.

As a consequence, by organizing these different fragments of memory, dreams could give rise to new connections and form a novel “story” that we have not experienced before, even exhibit a scene that never happens in real life. However, memories which will be weaken or faded away may be also a component of dreams, so experiences that are selectively incorporated into dream content need more investigations.

## The strengths of self-organization theory of dreaming

Taking account of the process of memory consolidation based on the two-stage model, self-organization theory of dreaming can explain many findings about dreams. Now we summarize them as follows.

1. It explains the relationship between dreaming and memory consolidation. The procedure of memory consolidation during sleep is involved with reactivation, abstraction, and extraction, which results in fragments of memory. Then these fragments could be a source of dreams. When self-organization process occurs, these materials are gradually combined into a narrative. Hence, dreams seem to be a by-product of brain neural activity and reflect memory consolidation during sleep. This point is similar with the model of Murkar et al. (2014).
2. It offers an explanation why dreams are easy to forget. As a result of dreaming brain's self-organization under a weak control, the combination of fragments (i.e., dreams) is just a temporary pattern. Thus, when the dreamer wakes and the control of brain revives, this impermanent stable state will disappear in a short time, and then the forgetting of dreams happens.
3. Incongruity and discontinuity are universal in dream content, as the nature of dreams is a combination of fragments. Although the narrative of dreams is relatively continuous due to self-organization, it would not be a “perfect” story. Thus, these defective connections between fragments will be inevitably presented in dream content. In addition, other factors during sleep will also disturb its continuity, such as PGO wave (Kahn and Hobson, 1993; Kahn et al., 2000, 2002).
4. This theory seems more reasonable than the Continuity Hypothesis. The Continuity Hypothesis considers that the content of dreams reflects a replay of waking life experiences, but investigations reveal that not everything happens in real life will be presented in dreams. In addition, the parts of incorporation are usually fragments rather than a complete scene. Hence, waking life experiences are selectively incorporated in dream content, and this procedure could be interpreted by memory consolidation.
5. It could explain some of the distinctions between dreams of REM sleep and those of NREM sleep (see Hobson et al., 2000). Firstly, the frequency of dreaming in REM sleep is higher than that in NREM sleep, because the brain's status of the former is more suitable for dreaming, such as neurochemical modulation, the activity level of prefrontal regions and limbic system, and PGO wave (Kahn et al., 2002). Secondly, the dream content of REM sleep is more novel and less associated with waking life compared with that of NREM sleep, as the processing of memories during REM sleep is more related to remote memories and usually makes new connections, while dreams of NREM sleep depend on the reactivation of newly encoded memories in waking life. Furthermore, the processing of different types of memory could also be a factor that leads to a difference of dream content between these two sleep stages.
6. From this perspective, the “simulation function” of dreams is merely a possibility that combines different information presented during sleep, and this “creative product” may provide an enlightenment to the dreamer. Several theories suggest that dreams may have simulation function, such as threat simulation (Revonsuo, 2000; Valli and Revonsuo, 2009), social interaction simulation (Brereton, 2000; McNamara et al., 2005) and protoconsciousness simulation (Hobson, 2009). Therefore, these researchers consider that dreams are an adaptive strategy for the individual, as it could increase the possibility of survival or develop mental function. However, the self-organization theory implies that dreams may be not functional themselves.
7. Self-organization theory of dreaming could be compatible with various kinds of psychological and physiological changes during sleep. Except for memories and PGO wave, some other factors, such as emotions (e.g., Yu, 2007; Vandekerckhove and Cluydts, 2010; Malinowski and Horton, 2014) and external stimuli (e.g., Nielsen, 1993; Schredl et al., 2009; Bloxham and Durrant, 2014), could impact on the content of dreams. Therefore, it could also interpret special types of dreams (e.g., bad dreams, recurrent dreams, and nightmares). As all of these elements could be incorporated into dreams through self-organization mechanism, this theory provides a compatible framework that covers different sources of dreams.

## Conclusion

Self-organization mechanism could offer a compatible framework that integrates different elements (e.g., memories, emotions, and external stimuli) into dreams. The two-stage model of memory consolidation could provide a gist how memories are abstracted, exacted and stored during sleep, and thereby become a source of dreams. From this perspective, dreams are a by-product of corresponding neural activity in sleeping brain. They reflect the function of NREM sleep and REM sleep, but they seem not to be functional themselves (Wamsley, 2014).

However, although self-organization theory of dreaming is compatible with most of findings about dreams, it is not easy to test. Furthermore, the incorporation rate of waking experiences is still obscure. Thus, this viewpoint needs more empirical evidences to support and enrich.

## Author contributions

The author confirms being the sole contributor of this work and approved it for publication.

### Conflict of interest statement

The author declares that the research was conducted in the absence of any commercial or financial relationships that could be construed as a potential conflict of interest.
